# Effects of free-range and confined housing on joint health in a herd of fattening pigs

**DOI:** 10.1186/s12917-014-0208-5

**Published:** 2014-09-11

**Authors:** Pernille Engelsen Etterlin, Bjørnar Ytrehus, Nils Lundeheim, Eva Heldmer, Julia Österberg, Stina Ekman

**Affiliations:** Section of Pathology, Department of Biomedical Sciences and Veterinary Public Health, Swedish University of Agricultural Sciences (SLU), 75007 Uppsala, Sweden; Terrestrial Ecology Department, Norwegian Institute for Nature Research (NINA), Trondheim, Norway; Department of Animal Breeding and Genetics, Swedish University of Agricultural Sciences (SLU), Uppsala, Sweden; Swedish Animal Health Service (SvDHV), 464 32 Mellerud, Sweden; National Veterinary Institute (SVA), 751 89 Uppsala, Sweden

**Keywords:** Pigs, Housing, Organic, Free-range, Joints, Osteochondrosis, Elbow, Hock

## Abstract

**Background:**

Free-range housing, in which pigs have access to both indoor and outdoor areas, is mandatory in organic pig production in Europe, but little is known about the effects of this housing on joint health in pigs. A high level of joint condemnations at slaughter has been reported in organic free-range pigs in Sweden, compared with pigs raised in conventional confined housing. We hypothesised that biomechanical forces imposed on the joints of pigs that range freely promote the development of osteochondrosis and lead to joint condemnation. We compared the prevalence of osteochondrosis and other joint lesions (e.g. arthritis, traumatic) in the elbow and hock joints of 91 crossbred Hampshire (Yorkshire × Landrace) fattening pigs that were housed in a free-range indoor/outdoor system with that in 45 pigs housed in confined indoor pens.

**Results:**

A larger proportion of free-range than confined pigs had osteochondrosis in the elbow joints (69 vs. 50%, *p* < 0.05), and a higher proportion of these joints in free-range pigs showed moderate or severe lesions (33 vs. 16%, *p* < 0.05). The free-range pigs also showed a higher prevalence of osteochondrosis in the hock joints (83 vs. 62%, *p* < 0.05) and a larger proportion of these joints had moderate or severe lesions (69 vs. 33%, *p* < 0.001). At slaughter, 4.2% of the free-range pigs had condemned joints, all of which showed severe osteochondrosis, while no joints of confined pigs were condemned.

**Conclusions:**

In this experiment the prevalence of osteochondrosis in the elbow and the hock was higher, and lesions were more severe, in free-range than in confined pigs, suggesting that free-range housing increases the risk of acquiring osteochondrosis. Increased biomechanical stress to vulnerable joint structures may be the mechanism behind this effect, however more studies are needed to verify these results. This study suggests that modification of housing, and breeding for joints that are more adapted to free-range movement may be needed in free-range pig production. Severe osteochondrosis is a cause of joint condemnation, but the condemnation rate at slaughter underestimates the actual frequency of joint lesions and hence is a poor assessment of joint health.

## Background

The number of organic fattening pigs slaughtered in Sweden rose from 3,000 in 1997 to 31,260 in 2012 [1]. The number of joints rejected at slaughter has also been rising. Meat inspection statistics collected by the Swedish Animal Health Service (SvDHV) over the last 16 years show that joints from 2.5 to 6.5% of all organic fattening pigs, compared to 1 to 2.2% of all joints from conventional pigs, are condemned at slaughter.

Previous studies on organic fattening pigs in Sweden indicate that osteochondrosis [2] and infectious conditions, mainly arthritis caused by *Erysipelothrix rhusiopathiae* [3], are the causes of lesions in the majority of condemned joints. However, there is a dearth of information on the normal health status of joints of fattening pigs in organic production. Joint condemnation rates are often mentioned in research and discussions on health in organic pigs [3-11], but it is unknown how well joint rejections at slaughter reflect the actual frequency of lesions in the joints of fattening pigs.

Osteochondrosis (OC), defined as a local disturbance in the process of endochondral ossification [12], occurs in all common pig breeds [13] and is an important cause of leg weakness and joint illness [14-17]. The primary lesions are ischemic necroses of growth cartilage due to premature cessation of the cartilage canal blood supply [18]. Lesions range from clinically silent, microscopic changes called osteochondrosis latens, to macroscopically visible retained cartilage called osteochondrosis manifesta, to osteochondrosis dissecans (OCD) [18-20], in which clefts form between the articular surface and the subchondral bone, causing joint inflammation [16,18,21,22]. Heredity, anatomical features, rapid growth, and nutrition are some of the most commonly discussed etiologies of OC [16,18]. Increased biomechanical stress and/or trauma have also been proposed as factors in the formation of primary lesions and the successive stages of OC [22-26].

A key characteristic of organic pig production is an enriched environment that includes a large space, free access to an outdoor paddock and/or pasture, deep straw bedding, and large group housing, all of which together provide a more ‘natural’ habitat for the pigs. Allowing pigs to range freely enables them to engage in various exploratory activities and exercise. Such an increase in activity presumably increases the magnitude and the diversity of biomechanical forces exerted on the joint structures. These forces may lead to increased incidence of microtrauma that causes failure of the cartilage canal blood supply and initiates the formation of osteochondrosis latens lesions, and to the progression of a manifest lesion to osteochondrosis dissecans, affecting both the prevalence and the severity of lesions.

The aim of this study was to describe the joint lesions and examine their frequency, and to compare the prevalence and severity of OC in fattening pigs raised in a free-range environment or a confined system located on the same farm. Furthermore, we suspected that conventional meat inspection fails to identify all joints with severe pathological lesions and we compared the condemnation rate with the true prevalence of lesions in two commonly affected joints.

## Methods

This study was approved by the Gothenburg Ethical Committee on Animal Research and was performed in the summer and autumn of 2012.

### Animals and housing

The study included 150 crossbred (offspring of Landrace × Yorkshire sows, inseminated with Hampshire semen) pigs born in the same week in one piglet-producing herd in Sweden, managed according to the European Union organic regulations on organic farming [27]. Piglets were housed with their sows in individual standard indoor Swedish farrowing pens (6 m^2^) until 2 weeks of age. The sows and their litters were then placed in two large adjacent group pens with deep straw bedding, from which they had access to an outdoor run with a concrete floor. After the piglets were weaned at approximately 7 weeks of age, the sows were removed from the pens. When they were twelve weeks old, 150 piglets were selected at random from these two pens and ear-tagged with identification numbers in both ears. The marked individuals were transported 160 km by truck to a commercial organic fattening farm in Sweden and subdivided into two different housing systems.

Fifty pigs were first unloaded and placed in confined indoor housing, in which five to seven pigs shared each 12-m^2^ pen. The pens had solid concrete floors, minimal bedding in the resting area, and a slatted concrete floor in the defecation area. The remaining 100 pigs were randomly sorted into two groups of 50 in a separate building and housed in two identical neighboring pens that complied with the EU regulations on organic farming [27]. Each pen had a 90-m^2^ indoor area that included a feeding area with a solid concrete floor, a resting area with deep (0.5 m) straw bedding, and a defecation area with a slatted concrete floor. The outdoor area consisted of a run with a concrete floor (26 m^2^) and access to pasture (approximately 2500 m^2^), as required by KRAV, the Swedish organic certification organisation [28]. The original experimental design called for only one of the two free-range pens to have access to the pasture. However, the barriers between the free-range pens were inadequate; the pigs intermingled from the first day and were thus considered as a single experimental group.

All pigs were provided water ad libitum and received the same feed. Pigs were fed three times each day in a feeding trough. The type and the amount of feed followed the specifications of the Swedish University of Agricultural Sciences (SLU) feeding norm [29] and the standards in the EU regulations on organic farming [27]. The pigs were fed almost ad libitum until they reached 60 kg average live weight, after which they received the average equivalent of 34 MJ per pig each day until slaughter. The farmer inspected the animals’ health daily, and we examined each pig individually on three separate occasions. Slaughter-ready pigs (5.5–6.5 months, approximately 100–110 kg live weight) from all housing groups were culled weekly and transported together 187 km to a large commercial slaughterhouse. Pigs that were euthanised because of clinical illness, lost their identification tags, or reached slaughter weight after 7 months of age were excluded from the study.

A pilot study on condemned joints in organic fattening pigs [2] showed that the hock and elbow joints were the most commonly affected. Therefore, our investigation focused on lesions in these two joints. After slaughter, all elbow and hock joints of all legs, including joints from condemned limbs, were collected unopened. The joints were separately packed, labelled and transported in refrigerator trucks to SLU in Uppsala, where they were frozen at -20°C until further analysis.

### Post-mortem examination

The joints were thawed, sampled, and examined at room temperature. Joints were opened using sterile techniques and the synovial fluid and membranes were assessed macroscopically for signs of inflammation. When joints showed discolouration, increased quantities of synovial fluid, or hyperemia or thickening/proliferation of the synovial membrane, samples were taken from three separate, standardised areas of the synovial membrane and immersed in 10% neutral buffered formalin. In addition, samples of synovial membrane were frozen for later research. The formalin-fixed synovial membranes were embedded in paraffin, dehydrated, cut into 3-μm sections, and stained with haematoxylin and eosin and examined histologically. Selected slides were Gram-stained.

The tissues surrounding the articulated bones were completely removed and the joint capsule was incised, separating the two bones. Assessment of pathological lesions was performed by the first author without knowledge of the identity or group allocation of the individual animals. The articular surfaces (distal humerus, proximal radius, proximal ulna, distal tibia, distal fibula, proximal and distal talus and calcaneus) and the intra-articular ligaments were subsequently inspected for lesions (ruptures, fractures, haemorrhages, or inflammation).

An initial examination of the presence or absence of OC, recognized as cleft formation, focal irregularity or whitening/thickening of the cartilage, was performed on all intact articular surfaces. A band saw was then used to section the epiphyses of the humeral condyle, talus, and calcaneus into 3-4 mm parallel slabs in a sagittal plane. A score for OC was assigned based on the macroscopic appearance of the cut surfaces of the slabs. The criteria for scoring OC lesions are provided in Table 1 and represent a modification of earlier proposed scoring systems [20,30] for expressing the severity of a given pathological process.

**Table 1:** **Grading scale for osteochondrosis lesions in the articular cartilage complex and subchondral bone tissue**

	**Score 1**	**Score 2**	**Score 3**	**Score 4**	**Score 5**
**Articular cartilage complex**					
Thickened cartilage (focal necrotic cartilage)*	**+**	**+**	**++**	**++**	**++**
Separation in osteochondral junction	**–**	**–**	**+**	**++**	**++**
Cleft(s) in articular cartilage	**–**	**–**	**+/–**	**+/–**	**++**
**Subchondral bone tissue**					
Hyperaemia, haemorrhage	**–**	**+**	**+**	**++**	**++**
Necrosis/fibrosis, subchondral cysts	**–**	**+/–**	**+**	**++**	**++**
**Extent of lesion**	Minor	Small	Moderate	Extensive	Severe

Lesions were assessed as minor–small (+), moderate–extensive (++), varying presence (+/**–**), or not present (**–**). A score of 0 reflected normal tissue with no osteochondral lesions; scores 1–5 indicated increasing size and number of lesions. *Lesions that were difficult to diagnose macroscopically were examined histologically for areas of necrotic cartilage.

The right and left joint were scored independently. If more than one osteochondral lesion occurred in the same location, the highest score was used for analysis.

All osteochondral lesions were photographed with a digital camera (Canon Rebel XTi Model DS126151, DC 8.1 V), and the scored slabs from each joint were immersed in 10% neutral buffered formalin. In slabs where the score was unclear macroscopically, the score was decided microscopically. These slabs, and slabs from joints with lesions that were regarded as typical for osteochondrosis, were decalcified in 3.4% (w/v) sodium formate and 15.5% (v/v) formic acid, prepared on histological slides (5 μm), and stained with haematoxylin and eosin for microscopic evaluation.

### Statistical analysis

The data were analysed using SAS v. 9.3 (SAS Institute. Inc., Cary, NC). The highest score of each variable in the left and right joint of each animal was used for statistical analysis. In addition to calculating frequencies and odds ratios on raw data, Spearman rank correlations between the OC scores of the corresponding right and left joints were calculated from their residual values after applying a linear model including the fixed effects of group, sex, and the regression of carcass weight. We estimated the effects of housing and sex on binary response variables using the GENMOD Procedure (logit link function in SAS). The analysed binary variables were the presence of OC (score 0/score 1–5), the severity of OC (two categories, 0–2 or 3–5), and the presence of OCD (score 0–4/score 5) in specific locations. The statistical model included the effects of group, sex, and the regression of carcass weight. The interaction between group and sex was included and tested in the initial model but was not significant for any of the analysed traits so was eliminated from the final model. Least-square means (adjusted means of proportions) were estimated using the inverse link option. In addition, the presence of synovitis, kissing lesions (i.e. abrasions in the articular cartilage and sometimes in the underlying subchondral bone, seen opposite an OC lesion), and any pathological lesion (including OC) in any location of every joint were examined statistically. Significance was defined as *p* ≤ 0.05.

## Results

Fourteen pigs were excluded from the study; three free-range and four confined pigs lost both ear tags; three free-range pigs were euthanised because of illness (one each with low growth rate, chronic pneumonia, and purulent arthritis and osteomyelitis in an elbow joint) before reaching 20 weeks of age; three free-range pigs did not reach slaughter weight until after seven months of age; and one confined pig was excluded due to missing slaughter data. The remaining 136 pigs (91 free-range, 45 confined) were included in the analyses as they showed good health and needed no treatment for disease during the growth period.

We examined 272 elbow and hock joints (136 right and left). Due to the difference in numbers of free-range (91) and confined (45) pigs, the results are presented as percentages of lesions. Inspection of the distal medial and lateral talus and the mediodistal calcaneus was not performed for the first 32 pigs because of an oversight, so the results for these locations were obtained from 104 pigs.

### Osteochondral lesions

**Figure 1:**
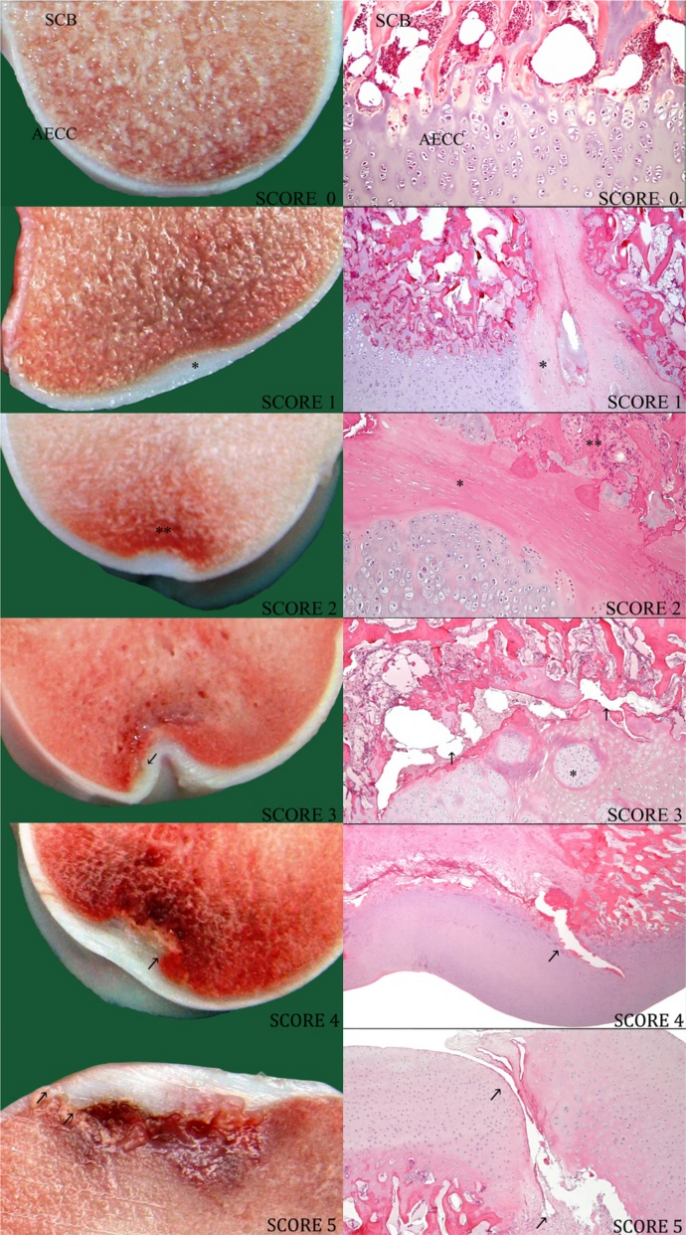
**Osteochondral lesions in the articular epiphyseal cartilage complex and subchondral bone of the talus.** These six macro- and microscopic images of slabs from the tali of different pigs show lesions that are characteristic of each osteochondrosis (OC) score. Together, they illustrate how lesions develops from minor OC (scores 1 and 2) through moderate to extensive OC (scores 3 and 4) and finally to OC dissecans (score 5). Open spaces in the microscopic images are artefacts. Score 0: No lesions in the articular epiphyseal cartilage complex (AECC) and subchondral bone (SCB) (medial distal talus, figure size × 10). Score 1: Minimal focal thickening* of the AECC with necrotic cartilage visible histologically (lateral distal talus, ×4). Score 2: Small focal thickening and necrosis in the AECC with vascular and/or inflammatory** reaction in subchondral bone (medial distal talus, ×10). Score 3: Moderate lesions in the AECC and underlying bone with minor separation (arrows) in the osteochondral junction. Focal chondrocyte clusters* are seen (medial distal talus, ×4). Score 4: Extensive lesions in the AECC and underlying bone where separation (arrows) is visible in both cartilage and subchondral bone (medial distal talus, ×1). Score 5: Osteochondrosis dissecans. Severe lesion with clefts that cut through the surface of the cartilage (arrows) with necrosis in the AECC and involvement of subchondral bone (medial trochlea of the talus, ×1).

The OC data are based on the scores from the slab examination. In total, 130 pigs had OC in at least one location in one or more joints. Typical OC lesions viewed macro- and microscopically on slabs are illustrated in Figure 1. OC lesions with score 4 or 5 showed depressions or irregularities at the surface of the articular cartilage, but the presence of these depressions varied in OC lesions with score 1, 2, or 3.

The presence and severity of osteochondral lesions in the joints of free-range and confined pigs are presented in Table 2 (elbows) and Table 3 (hocks).

**Table 2:** **Distribution (percentage) of osteochondrosis (OC) scores in the right and left elbow joints**

**Humeral condyle**	**Group**	**Joint**	**OC 0**	**OC 1**	**OC 2**	**OC 3**	**OC 4**	**OC 5**	**Total OC**
Medial aspect	Free-range	Right	60	10	10	4	7	9	40
		Left	58	9	6	9	9	9	42
	Confined	Right	71	9	9	4	3	4	29
		Left	71	9	13	5	0	2	29
Lateral aspect	Free-range	Right	78	16	6	0	0	0	22
		Left	80	14	3	0	1	2	20
	Confined	Right	73	18	2	7	0	0	27
		Left	76	18	2	2	2	0	24

The results are rounded. Number of examined animals: free-range = 91, confined = 45.

**Table 3:** **Distribution (percentage) of osteochondrosis (OC) scores in the right and left hock joints**

**Humeral condyle**	**Group**	**Joint**	**OC 0**	**OC 1**	**OC 2**	**OC 3**	**OC 4**	**OC 5**	**Total OC**
Medial talus trochlea	Free-range	Right	70	4	6	2	0	18	30
		Left	71	6	6	4	0	13	29
	Confined	Right	85	9	3	0	0	3	15
		Left	78	11	4	7	0	0	22
Medial talus distal*	Free-range	Right	47	3	16	28	4	2	53
		Left	43	6	19	28	4	0	57
	Confined	Right	61	3	19	17	0	0	39
		Left	61	8	17	11	3	0	39
Lateral talus trochlea	Free-range	Right	81	8	6	3	1	1	19
		Left	82	6	3	8	0	1	18
	Confined	Right	91	9	0	0	0	0	9
		Left	82	9	9	0	0	0	18
Lateral talus distal*	Free-range	Right	60	13	19	8	0	0	40
		Left	62	13	15	10	0	0	38
	Confined	Right	64	17	17	2	0	0	36
		Left	64	8	22	6	0	0	36
Calcaneus coracoid process	Free-range	Right	74	1	1	0	0	24	26
		Left	72	0	3	1	1	23	28
	Confined	Right	93	0	2	0	0	5	7
		Left	98	0	0	0	2	0	2
Calcaneus mediodistal*	Free-range	Right	75	7	16	2	0	0	25
		Left	75	6	15	4	0	0	25
	Confined	Right	88	6	6	0	0	0	12
		Left	80	6	14	0	0	0	20

The values are rounded. Number of examined animals: free-range = 91, confined = 45.

*These joint sites were examined in 68 free-range and 36 confined pigs.

#### Osteochondrosis in the elbow joints

The percentage of pigs with an OC score in any location of the elbow joint was 68.1% in the free-range group and 53.3% in the confined group (odds ratio 1.9, i.e. the odds of OC in the elbow was 1.9 times greater in the free-range than confined pigs). Pigs from both groups had greater number of and more severe lesions in the medial aspect of the humeral condyle than in the lateral aspect. The percentage of pigs that had an OC score of 3, 4, or 5 in any location of the elbow joint was 33% in the free-range group and 15.6% in the confined group (odds ratio 2.7).

Kissing lesions were apparent on joint surfaces opposite some of the larger OCD lesions in the medial humeral condyle in 4% of the right and 6% of the left elbow joints of free-range pigs, but were not found in any of the confined pigs. The kissing lesions affected areas of the semilunar notch, anconeus, and/or the proximal anterior ridge of the radius.

#### Osteochondrosis in the hock joints

OC lesions in the hock joint occurred in 82.4% of free-range and 64.4% of confined pigs with an odds ratio of 2.6. In both groups, OC was most prevalent in the distal medial and lateral parts of the talus. The severity of osteochondral lesions, calculated as the percentage of animals with an OC score of 3, 4 or 5 in any location, was 69.2% in the free-range group and 35.5% in the confined group (odds ratio 4.1).

The most common and severe lesions occurred on the medial side of the articular surface in both groups. However, in the free-range group, OC was also common in the coracoid process of the calcaneus. Of the 36 free-range pigs that had an OCD lesion in one or both hock joints, the lesion occurred in only the coracoid process in 17 animals and in both the coracoid process and the medial proximal trochlea in 10 animals. Only four pigs in the confined group had OCD, two in the coracoid process and two in the medial proximal trochlea. The osteochondral lesions in the lateral distal talus and mediodistal calcaneus were all mild to moderate (OC score of 1, 2, or 3) in all pigs.

**Figure 2:**
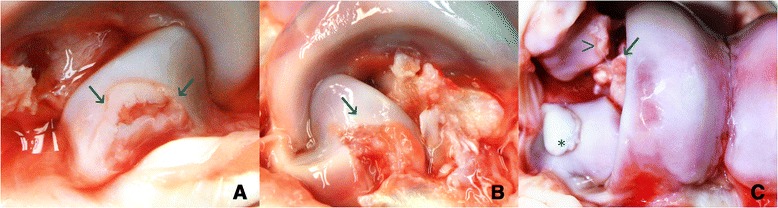
**Osteochondrosis dissecans lesions in the coracoid process of calcaneus. A**. Lateral close-up of the right coracoid process: multiple clefts in the articular cartilage expose underlying subchondral bone tissue and have caused synovitis with increased amounts of transparent/slightly haemorrhagic synovial fluid. **B**. Lateral view of the left coracoid process: a large fragment of articular cartilage has loosened from the subchondral bone tissue (arrow). The exposed bone and surrounding synovial and soft tissues are inflamed. **C**. Frontal view of the right lateral trochlea and coracoid process: osteochondral fragment (*) adherent on the surface of the coracoid process. Kissing lesions on the lateral malleolus of the fibula (arrowhead) and fascies articularis malleolaris of the talus (arrow) are also seen.

Lesions of the coracoid process were 2–15 mm in diameter with fragments of necrotic articular cartilage and single or multiple cleft formations, sometimes exposing underlying subchondral bone (Figure 2A and B). In a few of the cases with scores of 5/OCD we found clefts in the articular epiphyseal cartilage complex (AECC) and the cartilage had a yellowish discolouration. Histologically the AECC lacked obvious cartilage thickening and only a minor necrosis of cartilage and minor reaction in the subchondral bone was observed. Rare cases had large osteochondral fragments that were up to 0.8 mm in diameter and were associated with clefts of the articular cartilage (Figure 2C).

Kissing lesions were primarily observed on the articular surfaces of the lateral malleolus of the fibula and the distal tibia. Kissing lesions were common in free-range pigs and were found in 20.8% and 13.2% of pigs on the right and left articular surface of the distal tibia, respectively, and in 27.5% and 19.8% of pigs on the right and the left lateral malleolus of the fibula, respectively. Only 4.4% of confined pigs had kissing lesions on the right distal tibia, and 2.2% of confined pigs had them on the right lateral malleolus of the fibula.

When OCD was present only in the coracoid process, the kissing lesions occurred on the lateral malleolus of the fibula. When OCD occurred in the medial or lateral proximal trochlea of the talus, there were always lesions on the articular surfaces of the lateral malleolus of the fibula and the distal tibia as well.

#### Assessing osteochondrosis on articular surface compared to slab examination

The percentage of OC recognized by inspection of intact joint surfaces was compared to the results recorded by examination of slabs to determine how many of the OC lesions were missed or misdiagnosed by relying on surface examination alone. All OC lesions with scores of 4 or 5 were apparent by surface examination but the majority of OC lesions with score 1, 2, or 3 were only diagnosed by slab examination. Only 37% of the OC lesions in the medial part and 15% of the OC lesions in the lateral part of the humeral condyle diagnosed on slab examination were recognized by surface inspection. In the hock, none of the OC lesions revealed by slab examination of the mediodistal calcaneus were recognized on the intact joint surface. In the other anatomical locations (the trochlea and distal aspect of the medial and lateral talus and the coracoid process of calcaneus) of the hock joint, surface inspection recognized 74%, 76%, 62%, 38% and 93% respectively, of the OC lesions seen on slab examination.

Small irregularities and depressions occasionally seen in the articular surfaces were occasionally mistaken for OC lesions on surface inspection, but in these cases the slabs and histology of these lesions showed no visible underlying pathological lesions.

### Synovitis

Non-purulent chronic synovitis was found mainly in the presence of OCD (in 9.4% of the elbow joints and 30.7% of the hock joints in the free-range pigs, and in 3.3% of elbow joints and 3.3% of hock joints in the confined group). This condition was also seen in a few free-range pigs that did not have OCD (in 0.82% of the elbow and hock joints), but was not seen in any OCD-free confined pigs.

#### Synovitis and OCD

Synovitis that was histologically characterised by synovial cell hyperplasia and hypertrophy, and by the infiltration of low numbers of scattered lymphocytes and plasma cells, occurred in all joints with OCD. However, the degree of inflammation varied according to the size and location of the OCD lesion. All OCD lesions in the medial and lateral part of the humeral condyle or in the trochlea and distal parts of the talus occurred in association with synovitis and presented a moderate to substantial increase in serosanguinous synovial fluid, which sometimes contained small quantities of fibrin. These joints were all visibly swollen. Most OCD lesions in the coracoid process were associated with a local hyperemic reaction of the synovial. Only 21% of joints with a solitary OCD lesion in this location had marked synovitis and visible swelling.

#### Non-purulent synovitis without OCD

One free-range pig had bilateral non-purulent synovitis with no OC lesions in the right elbow joint and an OC score of 1 in the lateral aspect of the condyle of the left elbow joint. In both locations, the synovitis was characterised by hypertrophic and hyperemic villi with marked neovascularisation and infiltration of lymphocytes, plasma cells, and macrophages in the subsynovial connective tissue. In another case, a right hock joint with an OC score of 2 also had chronic proliferative synovitis with marked mature granulation tissue. Gram staining did not reveal bacteria in any of these cases.

### Other lesions

Other lesions were recorded only in elbow joints and were seen equivalently in free-range and confined pigs. These lesions included two joints with large haemorrhages in the capsule and synovial membrane. Four joints with partial ruptures and haemorrhages and three joints with minor haemorrhages were also observed in the collateral ligaments at their origin on the humerus. Seven joints had minor- to medium-sized fractures in the anconeus growth plate, with mild to moderate inflammation in the subchondral bone and synovial membrane.

### Effects of housing, sex, and weight on the prevalence and severity of osteochondrosis

Statistical results estimated by least squares analysis (GENMOD) for groups and sex are presented in Table 4 (elbow) and Table 5 (hock).

**Table 4:** **Estimated least squares means by group and sex for elbow joint lesions**

**Location**	**Lesion**	**Group**			**Sex**		
		**Free-range**	**Confined**	**Sign.**	**Gilt**	**Castrate**	**Sign.**
**Humeral condyle medial aspect**	OC 1–5	0.55	0.36	n.s.	0.37	0.54	n.s.
	OC 3–5	0.32	0.12	**	0.16	0.24	n.s.
	OCD	0.11	0.05	n.s.	0.05	0.11	n.s.
**Humeral condyle lateral aspect**	OC 1–5	0.31	0.28	n.s.	0.41	0.21	*
	OC 3–5	0.02	0.04	n.s.	0.07	0.02	n.s.
	OCD	0.00	0.00	n.s.	0.00	0.00	n.s.
**All locations**	OC 1–5	0.69	0.50	*	0.63	0.58	n.s.
	OC 3–5	0.33	0.16	*	0.21	0.25	n.s.
**Synovium**	Synovitis	0.15	0.04	n.s.	0.06	0.10	n.s.
**All locations**	Any lesion	0.68	0.52	n.s.	0.65	0.56	n.s.

Measures of prevalence (score 1–5) and severity (score 3–5) of osteochondrosis (OC) and prevalence of osteochondrosis dissecans (OCD), synovitis, and any lesion (OC + all other lesions in the joint). Significant (Sign.) differences between groups and sex are indicated by asterisks *p ≤ 0.05; **p ≤ 0.01; n.s.; non-significant.

**Table 5:** **Estimated least squares means by group and sex for hock joint lesions**

**Location**	**Lesion**	**Group**			**Sex**		
		**Free-range**	**Confined**	**Sign.**	**Gilt**	**Castrate**	**Sign.**
**Medial talus trochlea**	OC 1–5	0.41	0.24	n.s.	0.29	0.35	n.s.
	OC 3–5	0.26	0.12	n.s.	0.15	0.21	n.s.
	OCD	0.17	0.05	*	0.10	0.08	n.s.
**Medial talus distal**	OC 1–5	0.78	0.47	**	0.52	0.74	*
	OC 3–5	0.49	0.25	*	0.34	0.38	n.s.
	OCD	0.00	0.00	n.s.	0.00	0.00	n.s.
**Lateral talus trochlea**	OC 1–5	0.28	0.21	n.s.	0.31	0.19	n.s.
	OC 3–5	0.13	0.00	***	0.00	0.00	n.s.
	OCD	0.00	0.00	n.s.	0.00	0.00	n.s.
**Lateral talus distal** ^^^	OC 1–5	0.58	0.42	n.s.	0.37	0.63	*
**Coracoid process**	OC 1–5	0.35	0.09	***	0.22	0.16	n.s.
	OC 3–5	0.32	0.07	***	0.19	0.13	n.s.
	OCD	0.31	0.04	***	0.15	0.10	n.s.
**Calcaneus mediodistal** ^**^**^	OC 1–5	0.37	0.21	n.s.	0.25	0.32	n.s.
**All locations**	OC 1–5	0.83	0.62	*	0.72	0.76	n.s.
	OC 3–5	0.69	0.33	***	0.47	0.57	n.s.
**Tibia, distal**	Kissing lesion	0.18	0.05	*	0.11	0.08	n.s.
**Lateral malleolus**	Kissing lesion	0.28	0.02	***	0.09	0.09	n.s.
**Synovium**	Synovitis	0.39	0.07	***	0.21	0.16	n.s.
**All locations**	Any lesion	0.83	0.65	n.s.	0.76	0.74	n.s.

Measures of prevalence (score 1–5) and severity (score 3–5) of osteochondrosis (OC) and prevalence of osteochondrosis dissecans (OCD), synovitis, and any lesion (OC and all other lesions in the joint). Significant (Sign.) differences between groups and sex are indicated by asterisks *p ≤ 0.05; **p ≤ 0.01; ***p ≤ 0.001; n.s.; non-significant. ^^^Only the difference in prevalence of OC was examined, as no cases of OC score 4–5 were recorded.

#### Free-range versus confined housing

Free-range pigs had more severe OC in the medial aspect of the humeral condyle than confined pigs (*p* < 0.05), and the prevalence of OC in all locations of the elbow joints was significantly higher (*p* < 0.05) in the free-range pigs. In addition, free-range pigs had a higher prevalence of OC in the medial distal talus (*p* < 0.01) and the coracoid process (*p* < 0.001), and more severe OC in the lateral trochlea (*p* < 0.01), the medial distal talus (*p* < 0.05) and the coracoid process (*p* < 0.001) of the hock joints compared to the confined pigs. In the hocks, OCD was more prevalent in the medial trochlea (*p* < 0.05) and the coracoid process (*p* < 0.001) of free-range pigs, as were synovitis (*p* < 0.001) and kissing lesions in the distal tibia (*p* < 0.05) and lateral malleolus of the fibula (*p* < 0.001). Osteochondrosis in the medial trochlea of the talus and the coracoid process contributed the most to the higher overall prevalence (*p* < 0.05) and severity (*p* < 0.001) of OC in the hock joints of the free-range pigs.

#### Sex

The free-range group included 37 castrated pigs and 54 gilts, the confined group included 22 castrated pigs and 23 gilts. The prevalence of OC in the lateral aspect of the humeral condyle of the elbow joints was higher (*p* < 0.05) in the gilts than in the castrates, while castrates had a higher prevalence of OC in the medial and the lateral distal talus (*p* < 0.05). The sexes showed no significant differences in OC in the other locations.

#### Weight

The mean (±SD) slaughter weight of free-range and confined pigs was 91.7 ± 5.3 and 96.0 ± 7.2 kg, respectively, and free-range pigs were slaughtered 6 ± 3 days later than confined pigs. Slaughter weight did not have a significant effect on the prevalence or severity of OC in any location of either joint.

### Correlations between OC scores in specific locations

No significant differences in the prevalence of OC were found between the left and right sides of either joint. Weak to moderate (*r* = 0.32-0.61) but significant (*p* < 0.01) associations between the left and right OC scores were noted for most locations (Table 6, elbow; Table 7, hock). Weak to moderate (*r* = 0.24-0.48, *p* < 0.01) correlation were also registered between the left and the right OC scores of the coracoid process of the calcaneus and the medial, respectively the lateral trochlea of talus.

**Table 6:** **Spearman rank correlations (r) between osteochondrosis (OC) score in right and left elbow joint**

		**OC score left humeral condyle**	
	**Location**	**Medial aspect**	**Lateral aspect**
**OC score right humeral condyle**	Medial aspect	0.52***	-0.04
	Lateral aspect	-0.10	0.52***

Significance levels: ***p ≤ 0.001.

**Table 7:** **Spearman rank correlations (r) between osteochondrosis (OC) score in right and left hock joint**

			**OC score left hock**					
	**Location**		**Medial talus**		**Lateral talus**		**Calcaneus**	
			**Trochlea**	**Distal**	**Trochlea**	**Distal**	**Coracoid process**	**Mediodistal**
**OC score right hock**	**Medial Talus**	Trochlea	0.45***	-0.04	0.34***	-0.07	0.36***	-0.02
		Distal	-0.05	0.32***	0.09	0.30**	-0.11	0.03
	**Lateral Talus**	Trochlea	0.26**	-0.04	0.15	-0.03	0.24**	-0.02
		Distal	0.09	0.06	0.00	0.46***	-0.04	0.20*
	**Calcaneus**	Coracoid process	0.24 **	-0.01	0.48***	0.09	0.61***	0.01
		Mediodistal	-0.12	0.18	-0.03	0.11	-0.01	0.49***

Significance levels: *p ≤ 0.05, **p ≤ 0.01, and ***p ≤ 0.001.

### Joints condemned at slaughter

The pathoanatomical examination showed that a total of 41 joints (elbow and hock joints included) had OCD with obvious synovitis (large amounts of synovial fluid and proliferated synovial membrane). The abattoir condemned five joints (one left and one right elbow joint and two right and one left hock joint) from four free-range pigs at slaughter, representing 1.4% of all joints (5 out of 364) and 4.4% of all pigs (4 out of 91) in the free-range group. No joints from confined pigs were condemned at slaughter. All the joints were condemned unopened and classified as arthritis/osteoarthritis by the meat inspectors. All the joints displayed OCD (score 5) and synovitis. Three of the condemned joints occurred in pigs that also had OCD in the opposite joints.

## Discussion

We found a significantly higher prevalence and severity of OC lesions in the elbow and hock joints of fattening pigs that were allowed to range freely, compared to pigs kept in confined housing, and these differences were most pronounced in the hock joints. These findings indicate that this disease may represent a larger health and animal welfare problem in free-range pig production than was previously assumed.

A higher prevalence of chronic joint lesions [31] and lameness [32-34] has been reported in fattening pigs housed in free-range or ‘animal-friendly’ systems with outdoor runs or pasture than in fattening pigs housed in confined indoor systems. However, Cagienard et al. [35] found no differences in clinical lameness or prevalence of swollen joints in animals in ‘animal friendly’/free range and traditional/confined housing systems. Others [31,36] have suggested that the incidence of leg disorders in free-range pigs may be underestimated because of the difficulties in diagnosing individual pigs that are group-housed in large systems. Lameness can be caused by various lesions; none of the above-mentioned studies included post-mortem disarticulation and examination of the joints. However, OC is often associated with leg weakness/lameness in pigs [15,16,22,37-41] and horses [42-44], and it is likely that OC lesions contributed to the lameness and swollen joints reported in previous studies.

### Housing factors

All pigs were crossbred Hampshires born and reared in the same herd prior to allocation into two different housing regimes at the age of 12 weeks. During the fattening period, the pigs received identical feed, according to the same feeding protocol and were handled by the same personnel. These facts strongly suggest that the housing environment from the age of 12 weeks to slaughter was an important explanation for the difference in prevalence and severity of OC between the free-range and confined pigs.

Grevenhof et al. [45] reported a lower prevalence of OC in the elbow and tarsocrural joints but a higher prevalence of OCD (‘score E’) in fattening pigs that were housed indoors on deep straw bedding with a larger space allowance than in pigs housed in confined stalls. Other studies failed to find significant associations between the prevalence of OC lesions and the type of flooring [46-48], and high stocking density was reported to have a significant effect on the prevalence of OC in only one location in the elbow [46]. However, the evaluation of osteochondral lesions in these studies was limited to gross inspection of intact joint surfaces. Here, we found that examination of thin, cross-sectioned joint slabs revealed a much higher prevalence of OC than did inspection of intact joint surfaces. Therefore, the prevalence of OC is likely to be underestimated in studies that use superficial scoring of joint surfaces, and differences among treatments in such studies might not be captured.

A comparative study between outdoor and conventionally housed indoor fattening pigs found that outdoor pigs walked more and were more active overall [49]. We did not measure activity in this study, but a main difference between the two housing systems was likely to have been the amount and type of activity among the pigs. Pigs in confined pens spend up to 80% of their time lying down [16], whereas pigs in an enriched environment are more active [50-53] and spend more time on exploratory activities, running, and jumping. Greater activity levels inherently result in a greater magnitude and diversity of biomechanical forces exerted on the joint structures. There are similarities between the forms of osteochondrosis that occur in humans and animals [54]; in humans, increased physical activity appears to promote the development of OC/OCD, either through biomechanical stress or repetitive trauma [55,56]. Activity has also been proposed as a cause of high prevalence of OC and lameness in joints of pigs in outdoor herds [57]. Other studies have shown that indoor exercise has positive effects on muscles and bones, improves locomotor ability [58], or has no effect on prevalence and severity of OC [58-60]. However, these studies used relatively small samples and in two of them bone slabs were not examined. Therefore, it is difficult to compare those results with the findings presented here.

Rather than the quantity of exercise, the nature of the rearing environment and the types of activity promoted by that environment may be the key factors that determine the overall biomechanical load on joint structures. The initial step in the formation of OC lesions in pigs and horses is thought to be microtrauma to the vasculature associated with the epiphyseal growth cartilage [18,19,23,24,61,62]. The risk of microtrauma is likely to be substantially higher in pigs that are allowed free-range movement than in confined animals. In addition, acute injury (‘macroscopic trauma’) may contribute to the progression from osteochondrosis manifesta to osteochondrosis dissecans [18,22,25].

In the present study, numerous environmental factors differed between the two housing groups, some of which could have increased the prevalence of irregular strain and repeated trauma on the joints of the free-range pigs, resulting in the differences in prevalence and severity of OC. Free-range pigs were housed in large groups with a large roaming area, variable flooring and soil hardness, uneven and stony pasture, and a high threshold between the deep straw bed and the feeding area. This threshold varied between 50 and 125 centimeters depending on the straw level and appeared to be increasingly difficult for the pigs to negotiate as their body mass increased. These housing factors might have had individual or interacting influences on the biomechanical stressors experienced by free-range pigs, and consequently on the development of OC. We do not have sufficient data to distinguish which factors were most important in this study. Additional studies involving more free-range farms are needed to examine the role of activity and the effects of various housing factors on the prevalence of OC in pigs.

### Osteochondrosis in the elbow and hock joints

Comparison of OC results obtained by inspection of intact surfaces and joint slabs showed that examination of intact articular surfaces would not identify many OC lesions with scores of 1, 2, or 3. Hence, we will limit our discussion to the results of the slab inspection.

The major difference in OC in the elbow joints of the two groups was that the free-range pigs had a significantly higher prevalence and more severe lesions in the medial aspect of the humeral condyle. The prevalence of OC and OCD in the medial aspect of the humeral condyle were within the ranges (OC, 6%–83.5%; OCD 1%–19%) reported in other studies of fattening pigs [38,45,63-66]. The prevalence and the severity of OC in both groups were higher in the hock joints than in the elbow joints. Grevenhof and colleagues [45] examined multiple joints and also found the highest prevalence of OC in the hock joint. High disease incidence (up to 76%) in the hock have also been reported in other studies [21,22,30].

We did not find previous reports on lesions in the mediodistal calcaneus; these lesions were only observed in slab sections and thus are easy to overlook. Osteochondral lesions in the coracoid process in fattening pigs have also not been reported previously, which may be explained by low incidence of this type of lesion in confined pigs, evident also in the present study. The coracoid process is of interest because the largest difference in prevalence and severity of OC between free-range and confined pigs was found at this lateral location. Previous reports have suggested that medial rather than lateral locations are prone to OC [16,18,22]. Furthermore, score 1–4 lesions were uncommon in the coracoid process and a variation in the extent of lesions present in score 5 lesions in this location indicated that the underlying pathological processes leading to clefts in the AECC might differ. Score 5 lesions that lacked substantial cartilage necrosis and thickening of the AECC might represent foci of OC that formed early in the life of the pig. After formation, the primary lesion might have resolved or been repaired, leaving only the cleaved joint cartilage as a visible manifestation. Because the majority of the lesions in the coracoid process were located on the most exposed area (the dorsolateral border), it is also possible that blunt acute trauma could have initiated some of the lesions in this location. Depending on its severity, trauma may only affect (i.e. cleave) the superficial articular cartilage or it may impair the blood supply in the cartilage canals of the AECC and so initiate osteochondrosis.

A central question is why an increase in presumably natural behaviour within presumed physiological limits should cause an increase in the prevalence and severity of osteochondrosis. Grøndalen [26] proposed that a certain conformation of the stifle joint causes local overloading in an area of the femoral condyle, which results in OC. Combining this information with current understanding of the pathogenesis of OC [18], we suggest that modern pigs, bred for convenient confined housing, have acquired traits that affect joint conformation and/or joint motion and that lead to unaccustomed physiological stress in certain locations of the joint, even during normal activity and motion.

Osteochondrosis manifesta (score 1–4) may develop into OCD (score 5) under the influence of various factors, one of which is mechanical load [18]. Alternatively, score 1, 2, and 3 OC lesions can resolve if they are engulfed by the ossification front and transformed into bone, or may be repaired by replacement with fibrous tissue that later undergoes membranous ossification. In addition, necrotic cartilage retained in the subchondral bone could also develop into a pseudocyst with micro fractures [67]. We cannot predict how the osteochondrosis manifesta lesions observed here would have progressed had the pigs not been slaughtered. Longitudinal studies including radiology, CT, and MRI have been performed in horses [68] and have provided evidence for the potential healing of OC lesions.

### General health

Most of the pigs in the present study were healthy and there were no general health differences between the two groups. Infectious arthritis was a rare joint lesion in the studied pigs. The effects of high growth rate and high slaughter weight on the development of OC in pigs is often debated [16,18,20,22,52,65,69], but here, the free-range pigs had lower average slaughter weight and greater incidence of OC than the confined pigs. We did not calculate growth rates in this study. Lundeheim [15] suggested that pigs with severe OC may not thrive and may have poor appetites, which could contribute to lower weights at slaughter.

### Correlations between OC scores in specific locations

Osteochondrosis is thought to be bilaterally symmetrical [21,22], and many studies of OC in pigs have included either the left or right leg but not both. Our findings showed that side does not have a systematic influence on the prevalence of OC. However, there was only a weak or moderate correlation between scores of OC lesions in a given location in the left and the right joints, which indicates that examining both legs provides a more complete picture of the severity of OC in specific joints. A weak to moderate association between the OC scores of the left and the right coracoid process of calcaneus and the medial, respectively the lateral trochlea of talus, indicates that a factor common to all of these locations influences the development of OC. The distal surfaces of the tarsocrural joint are all affected by variation in movement and load of the right and left distal tibia/fibula, which could help to explain the association between OC scores in these locations.

These findings, and the fact that significant differences in the prevalence and severity of OC between free-range and confined pigs occurred in only some joint locations, support the assumption that local and temporal factors affect the progression of OC. These factors include biomechanical forces associated with increased magnitude and diversity of activity.

### Joint condemnations

The large number of joints that had OCD lesions with clear synovitis (41), and the very low number of condemned joints (5) clearly showed that inspection of unopened joints at slaughter fails to identify a considerable proportion of joints with pathological lesions. If joint condemnations captured those with synovitis/arthritis and OC scores of 4 or 5, condemnation rates could be used in large epidemiological studies as a simple estimate of joint health in fattening pigs. However, our results show that joint condemnations provide a poor estimate of the prevalence of joint lesions in fattening pigs. Therefore, epidemiological studies on joint health that contain conclusions based only on condemnation rates are likely to be of limited value.

## Conclusions

This study indicated that the type of housing environment from the age of 12 weeks to slaughter, allowing either a high or low level of motion and activity, has a substantial effect on the development of OC in pigs. The results support our hypothesis that the increased magnitude and diversity of biomechanical stress experienced by free-range pigs promotes the development of OC. Further research is required to confirm our findings and should focus on the role of activity and the ways in which housing variables (e.g. group size, roaming area, outdoor pasture, flooring, and thresholds) may influence the development of OC. Infectious arthritis was not an important cause of joint lesions in the studied pigs. The condemnation rate did not mirror the joint health in the studied population, and conclusions about joint health that are based on condemnation data are likely to be erroneous. Likewise, studies that use scoring of joint lesions after inspection of intact joint surfaces alone will likely fail to report the true prevalence of the lesions.

Many of the joint lesions observed here may cause pain and eventually lead to lameness [40-43]. Free-range housing systems are an essential part of the high animal welfare standards of organic production, providing an environment that allows the animals to express their innate behaviours. More research on the associations between osteochondrosis, lameness/gait, and environmental factors in free-range fattening pig production is needed.

The same breeds are used in organic and conventional pig production systems in Sweden [70], and these breeds have been developed to cope with conventional environments, not free-range housing systems. Breeding of robust pigs that maintain good joint and leg health in free-range environment should also be promoted.
